# Post-weaning social isolation alters sociability in a sex-specific manner

**DOI:** 10.3389/fnbeh.2024.1444596

**Published:** 2024-08-29

**Authors:** Teneisha Myers, Elizabeth A. Birmingham, Brigham T. Rhoads, Anna G. McGrath, Nylah A. Miles, Carmen B. Schuldt, Lisa A. Briand

**Affiliations:** ^1^Neuroscience Program, Temple University, Philadelphia, PA, United States; ^2^Department of Psychology and Neuroscience, Temple University, Philadelphia, PA, United States

**Keywords:** social isolation, adolescence, minocycline, social interaction, sex differences

## Abstract

Adolescence is a critical period for brain development in humans and stress exposure during this time can have lasting effects on behavior and brain development. Social isolation and loneliness are particularly salient stressors that lead to detrimental mental health outcomes particularly in females, although most of the preclinical work on social isolation has been done in male animals. Our lab has developed a model of post-weaning adolescent social isolation that leads to increased drug reward sensitivity and altered neuronal structure in limbic brain regions. The current study utilized this model to determine the impact of adolescent social isolation on a three-chamber social interaction task both during adolescence and adulthood. We found that while post-weaning isolation does not alter social interaction during adolescence (PND45), it has sex-specific effects on social interaction in young adulthood (PND60), potentiating social interaction in male mice and decreasing it in female mice. As early life stress can activate microglia leading to alterations in neuronal pruning, we next examined the impact of inhibiting microglial activation with daily minocycline administration during the first 3 weeks of social isolation on these changes in social interaction. During adolescence, minocycline dampened social interaction in male mice, while having no effect in females. In contrast, during young adulthood, minocycline did not alter the impact of adolescent social isolation in males, with socially isolated males exhibiting higher levels of social interaction compared to their group housed counterparts. In females, adolescent minocycline treatment reversed the effect of social isolation leading to increased social interaction in the social isolation group, mimicking what is seen in naïve males. Taken together, adolescent social isolation leads to sex-specific effects on social interaction in young adulthood and adolescent minocycline treatment alters the effects of social isolation in females, but not males.

## 1 Introduction

Since the COVID-19 pandemic, adolescents are experiencing social isolation in ways that they have not previously. The level of social isolation experienced during the height of the COVID-19 pandemic has resulted in higher rates of depression and anxiety among young adults (Zhou et al., 2020). Conversely, high quality interactions with peers during adolescence in humans promote resilience later in life (van Harmelen et al., 2017). Rodent models have also demonstrated that social isolation, whether brief or chronic, has long lasting effects on brain development and behavior into adulthood. In rats, prolonged social isolation results in increased aggression toward conspecifics (Popova and Petkov, 1990). These changes in behavior are reflected in changes to the hypothalamic-pituitary-adrenal (HPA) axis and subsequent release of stress related hormones such as corticosterone in rodents (Cacioppo et al., 2015). In mice, social isolation increases anxiety-like behavior and promotes ethanol seeking behavior (Kwak et al., 2009; Lopez and Laber, 2015). Additionally, these changes are more prominent in females than in males, as females experience higher rates of anxiety-like behavior than males following social isolation (Liu et al., 2020).

Post-weaning social isolation has effects far beyond behavior. Stressful experiences lead to changes in synaptic plasticity in various regions of the brain, and the brain is especially vulnerable to these changes during postnatal development (McGrath and Briand, 2019). Recent research has focused on microglia and their critical role in these changes. Microglia are a crucial part of the brain's immune system, and chronic stress leads to increased activation of this system. Microglial activation following chronic stress causes morphological changes to microglia in which they become hyper-ramified. Hyper-ramification of microglia following chronic psychological stress is associated with behaviors that are representative of anhedonia and anxiety (Tynan et al., 2010). Examining the effects of chronic stress, such as adolescent social isolation, is critical in understanding how the brain is permanently changed over time, and how these changes can be attenuated.

The anti-inflammatory drug, minocycline, has shown success in inhibiting microglial growth. Minocycline administration during a period of prolonged stress successfully reverses the effects of chronic stress exposure on microglial ramification (Hinwood et al., 2013). Inhibition of microglia through the administration of minocycline is not only effective in reversing the effects of stress, it also increases active coping behaviors in rodents (Schmidtner et al., 2019). Minocycline also encourages active recovery from social withdrawal (Henry et al., 2008). The current study aims to examine how minocycline administration impacts the effects of adolescent social isolation stress on social behavior both in adolescence and in adulthood.

## 2 Material and methods

### 2.1 Subjects

Male and female c57BL/6J mice were bred in house. On PND 21, at weaning, mice were randomly assigned to either isolation or group housing (3–5 mice per cage). Mice remained in these housing conditions for the duration of the experiment. All mice were held in an animal care facility with temperature and humidity control and kept on a 12 h light/dark cycle beginning at 7:30 a.m. The animals received food and water *ad libitum* and were given a cotton nestlet for enrichment. All procedures were approved by the Temple University Animal Care and Use Committee.

### 2.2 Drugs

Minocycline was purchased from Millipore Sigma (Burlington, MA) and was dissolved in sterile 0.9% saline. A dose of 40 mg/kg was delivered daily via intraperitoneal injection from postnatal day 21–42.

### 2.3 Three-chambered social interaction test

**Figure 1:**
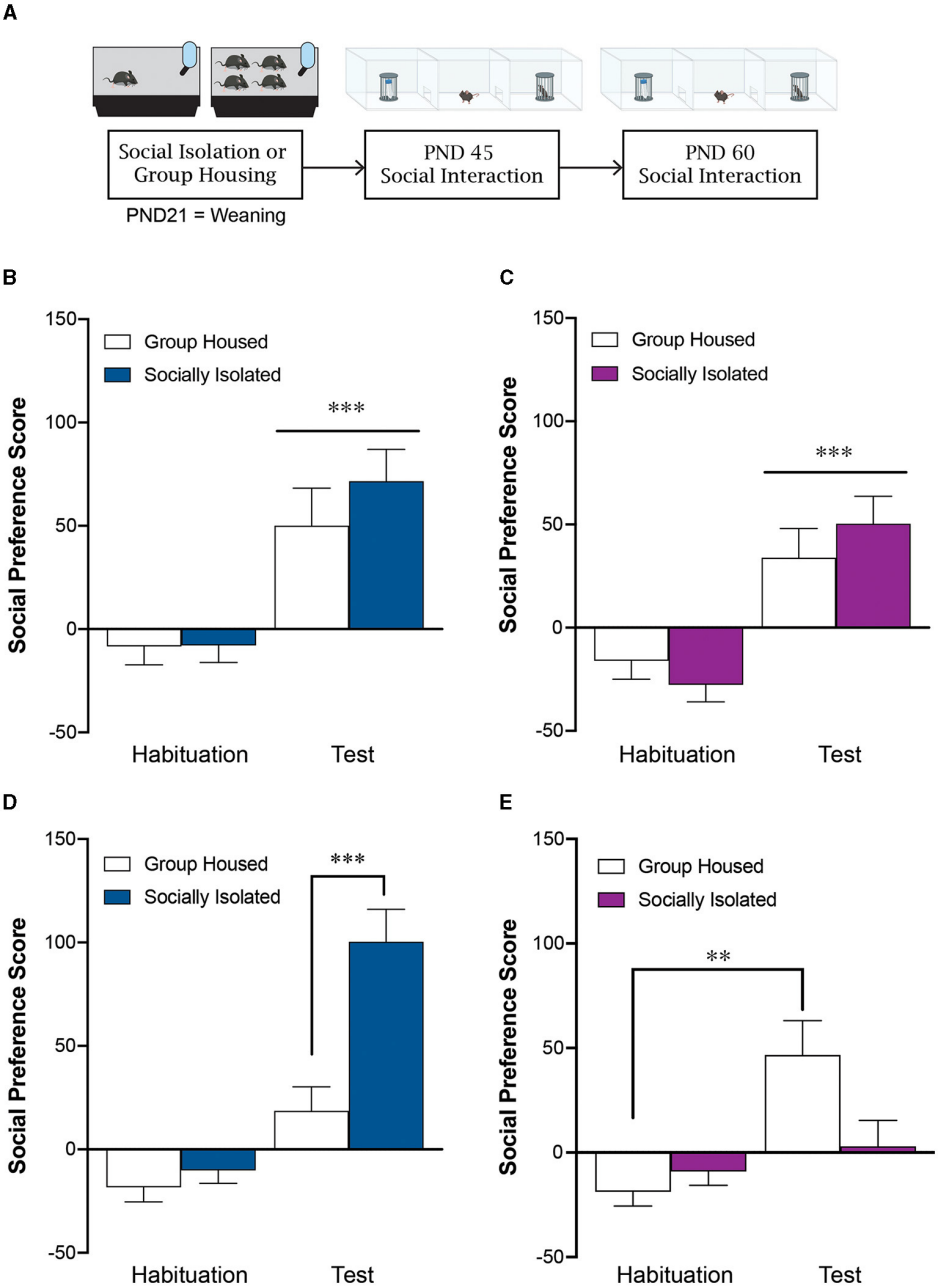
Post-weaning social isolation stress leads to sex-specific effects on social behavior during adulthood. **(A)** Experimental timeline. Post-weaning social isolation stress does not impact male **(B)** or female **(C)** social preference score at PND45. Post-weaning social isolation leads to a significant increase in social preference score in males **(D)** and a significant decrease in social preference score in females **(E)** at PND60. **p < 0.01, ***p < 0.001; *n* = 16–21/group.

All animals were tested twice in a three-chambered social interaction test, once in adolescence (PND45) and once in young adulthood (PND60). The apparatus consisted of a rectangular plexiglass three-chambered box with a lid. Each chamber was 16 × 8 × 9 in with a 2 × 3 in. (high) door leading from the center chamber to either right or left side. Both right and left sides of the chamber contained a wire cage measuring 3.5 in. in diameter and 7.5 in. tall for either a novel partner or novel object to be placed in. Novel partners were age and sex matched to the test animal and the partner and object used at PND60 were different than the partner and object used at PND45. After being brought from the animal facility, the animals were allowed to habituate for 20 min to the dimly lit room (93 lumens). In the first portion of the test, the animals were allowed to freely explore the apparatus for 20 min. Time spent interacting with the empty cylinders by sniffing was hand scored using AnyMaze tracking software. The animal was then briefly removed while the novel partner and novel object were placed in the cylinders. The side which contained the novel partner vs. novel object were randomized in a counter-balanced fashion. The animal was placed back into the middle chamber of the apparatus and allowed to freely explore for 10 min. Time spent interacting with the cylinders by sniffing was again hand scored using AnyMaze tracking software (Figure 1A).

### 2.4 Adolescent drug treatment

A subset of animals received daily intraperitoneal (I.P.) injections of minocycline or saline for 21 days starting at PND21. Mice were weighed daily at the time of injection to insure proper dosing. We did not observe a significant difference in social interaction between the unhandled animals compared to the saline animals [exploration time during habituation, *t*_(75)_ = 0.015, *p* = 0.98; exploration during social phase, *t*_(75)_ = 0.72, *p* = 0.47] therefore, the data from the unhandled and saline animals were combined.

### 2.5 Data analysis

Analyses were performed using GraphPad Prism 9.5 Software. All data from the social interaction experiments were analyzed using repeated measures two-way ANOVAs with test phase (habituation and test) as the repeated independent variable and housing condition as the second independent variable and social preference score as the dependent variable. Data were analyzed separately at each age (PND45; PND60). The social preference score was calculated by subtracting the time the experimental animal spent sniffing the novel object cylinder from the time the experimental animal spent sniffing the novel partner cylinder. For the habituation phase both cylinders are empty but the preference score is based on the preference for the cylinder that would contain the novel partner during the social phase of the test. To determine whether there were sex differences during young adulthood, additional two-way ANOVAs were run with only data from the test phase at PND60 and sex and housing condition as the independent variables. Sidak's *post*-*hoc* comparisons were made when interactions were detected (*p* < 0.05).

## 3 Results

### 3.1 Post weaning social isolation does not alter sociability in adolescence

Group housed (GH) and socially isolated (SI) male mice exhibit a similar social preference at postnatal day 45 (PND45) exhibiting a significantly higher social preference score during the test phase compared to the habituation phase [effect of test: *F*_(1, 38)_ = 23.2, *p* < 0.0001; effect of housing: *F*_(1, 38)_ = 0.774, *p* = 0.39; interaction: *F*_(1, 38)_ = 0.547, *p* = 0.46; *n* = 20/group; Figure 1B]. Similarly, at PND45, female mice exhibit a social preference regardless of whether they were group housed or socially isolated at weaning [effect of test: *F*_(1, 35)_ = 35.6, *p* < 0.0001; effect of housing: *F*_(1, 35)_ = 0.0359, *p* = 0.85; Interaction: *F*_(1, 35)_ = 1.77, *p* = 0.19; *n* = 16–21/group; Figure 1C].

### 3.2 Post weaning social isolation has sex-specific effects on sociability in young adulthood

During young adulthood on postnatal day 60 (PND60), the socially isolated males displayed significantly greater social preference than group housed controls [effect of housing: *F*_(1, 38)_ = 13.9, *p* = 0.0006; effect of test: *F*_(1, 38)_ = 60.13, *p* < 0.0001; interaction: *F*_(1, 38)_ = 14.9, *p* = 0.0004; *post-hoc* tests: GH test vs SI test: *p* < 0.0001; GH habituation vs. GH test: *p* = 0.02; SI habituation vs. SI test: *p* < 0.0001*; n* = 20/group; Figure 1D]. In contrast, adolescent social isolation decreased social preference in females at PND60 compared to group housed controls [effect of housing: *F*_(1, 35)_ = 7.12, *p* = 0.178; effect of test: *F*_(1, 35)_ = 46.1, *p* < 0.0001; interaction: *F*_(1, 35)_ = 4.29, *p* = 0.046; *post-hoc* test: GH test vs. SI test: *p* = 0.001; GH habituation vs. GH test: *p* =0.001; SI habituation vs. SI test: *p* = 0.79; *n* = 16–21/group; Figure 1E]. When we statistically compare the social preference score during the test phase across males and females we see a significant interaction between biological sex and housing condition [effect of housing: *F*_(1, 73)_ = 1.69, *p* = 0.198; effect of sex: *F*_(1, 73)_ = 5.65, *p* = 0.02; interaction: *F*_(1, 73)_ = 18.45, *p* < 0.0001; *post-hoc* test: male GH vs. male SI: *p* = 0.001; female GH vs. female SI: *p* = 0.04; male SI vs. female SI: *p* < 0.0001].

### 3.3 Adolescent minocycline treatment decreases sociability in males but not females

**Figure 2:**
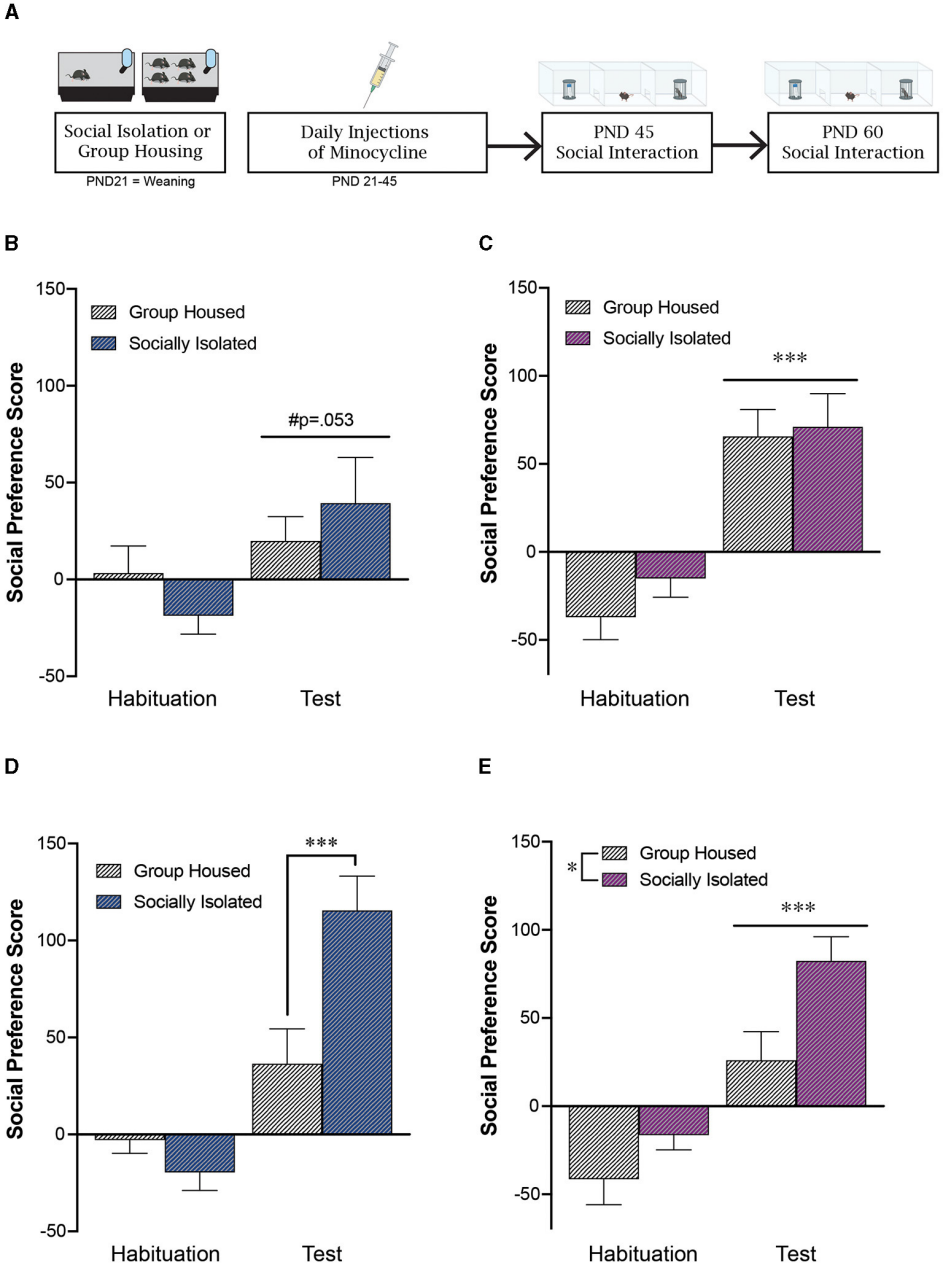
Adolescent minocycline treatment reverses the impact of post-weaning social isolation stress on social behavior in females during adulthood. **(A)** Experimental timeline. Adolescent minocycline treatment decreases social preference score in males **(B)** at PND 45, while not impacting social preference in females at this timepoint **(C)**. At PND60, adolescent minocycline treatment did not alter the impact of post-weaning social isolation, as isolated males continued to exhibit an increase in social preference **(D)**. In contrast, adolescent minocycline treatment reversed the impact of adolescent social isolation in females, with isolated females exhibiting an increase in social preference at PND60 compared to group housed females **(E)**. *p < 0.05, ***p < 0.001; *n* = 10–12/group.

Adolescent minocycline treatment dampened social preference in adolescent males at PND45 [effect of test: *F*_(1, 20)_ = 4.21, *p* = 0.053; effect of housing: *F*_(1, 20)_ = 0.007, *p* = 0.93; interaction: *F*_(1, 20)_ = 1.31, *p* = 0.27; *n* = 10–12/group; Figures 2A, B]. However, adolescent minocycline treatment did not alter social preference in females at PND45 [effect of test: *F*_(1, 19)_ = 34.8, *p* < 0.0001; effect of housing: *F*_(1, 19)_ = 1.07, *p* = 0.31; interaction: *F*_(1, 19)_ = 0.26, *p* = 0.61; *n* = 10–11/group; Figure 2C]. Of note, we did not detect any effect of minocycline in total exploration time spent sniffing during the habituation phase (MEAN±STDEV: drug naïve GH males: 154 ± 48 s; drug naïve ISO males: 126 ± 46 s; minocycline GH males: 135 ± 64 s; minocycline ISO males: 131 ± 23 s).

### 3.4 Adolescent minocycline treatment reverses impact of social isolation on sociability in females in young adulthood

Similar to what was seen in our unhandled and saline treated male mice, postweaning social isolation led to an increase in social preference in adult male mice compared to group housed controls [effect of housing: *F*_(1, 20)_ = 4.56, *p* = 0.045; effect of test: *F*_(1, 20)_ = 41.9, *p* < 0.0001; interaction: *F*_(1, 20)_ = 12.63, *p* = 0.002; *post-hoc* test: GH test vs. SI test: *p* = 0.0006; Figure 2D]. In contrast to what is seen in our unhandled and saline treated female mice, following adolescent minocycline treatment, both group-housed and socially isolated females exhibited a significant social preference [effect of housing: *F*_(1, 20)_ = 7.19, *p* = 0.015; effect of test: *F*_(1, 20)_ = 46.1, *p* < 0.0001; interaction: *F*_(1, 20)_ = 1.64, *p* = 0.22; Figure 2E]. In contrast to what we see in the absence of minocycline, following minocycline treatment, when we statistically compare the social preference score during the test phase across males and females we do not see a significant interaction between biological sex and housing condition [effect of housing: *F*_(1, 39)_ = 16.39, *p* = 0.0002; effect of sex: *F*_(1, 39)_ = 1.70, *p* = 0.20; interaction: *F*_(1, 39)_ = 0.46, *p* = 0.50].

## 4 Discussion

Overall, the present study demonstrates that post-weaning social isolation stress leads to a sex-specific effect on sociability during young adulthood. In male mice, adolescent social isolation potentiated social preference in young adulthood whereas in female mice social preference was disrupted. During adolescence, no effects of housing were seen suggesting that the social isolation is impacting a developmental process that occurs between PND45 and PND60. Along with these sex-specific effects of post-weaning social isolation, we also found sex differences in the ability of minocycline to alter these social phenotypes. While minocycline disrupted social preference in adolescent males regardless of housing condition, it had no impact on the ability of adolescent social isolation to increase sociability in young adult males. In contrast, minocycline reversed the direction of the effects of post-weaning social isolation on young adult social behavior in females, with minocycline treated females exhibiting increased sociability following adolescent social isolation. This data suggests a sex-specific role for microglia that differs over developmental time periods.

### 4.1 Post-weaning social isolation stress leads to sex-specific effects on social behavior during young adulthood

Adolescent social isolation leads to structural and functional changes in the brain (Orben et al., 2020; Deutschmann et al., 2022; McGrath and Briand, 2022). Chronic stress during this developmental period further increases one's susceptibility to neuropsychiatric diseases in adulthood, including anxiety, affective disorders, and alcohol and substance use disorders (Rivera-Irizarry et al., 2020). Many stress-related psychiatric diseases are more prevalent in women (Kessler et al., 2012; Altemus et al., 2014) and understanding the potential sex-specific impacts of adolescent stress can provide models to further explore the brain mechanisms underlying stress-induced vulnerability.

The present study investigated the effects of post-weaning social isolation on social behavior in males and females. We found that post-weaning social isolation led to potentiated social preference in male mice in young adulthood at PND60. Other groups have demonstrated increased social preference following post-weaning social isolation in males (Ferdman et al., 2007; Rivera-Irizarry et al., 2020). In addition, other models of post-weaning stress in male mice have also led to an increase in social preference in adulthood. Repeated footshock stress at PND21 led to an increase in social interaction in males in adulthood (Kumamoto et al., 2018). Although there is some congruence in the literature, not all studies examining the impact of post-weaning social isolation on social preference find consistent results (Kuniishi et al., 2022). These discrepancies may be impacted by the age at testing. In the current study we find no impact of social isolation at PND45 and studies that have examined social interaction at older time points (Ferdman et al., 2007; Rivera-Irizarry et al., 2020) are more likely to see increases is social interaction compared to those that look earlier (Kuniishi et al., 2022). Additionally, many of these other studies have examined a battery of behavioral tests including tests that elicit anxiety-like behavior (Rivera-Irizarry et al., 2020; Kuniishi et al., 2022) and the timing of these tests relative to the social interaction testing could influence these results (Lukkes et al., 2009). Differences could also be driven by differences in novelty preference as the social interaction procedure in the current study compared social interaction to exploration of a novel object rather than an empty cylinder or chamber as done in many other studies (Kumamoto et al., 2018; Rivera-Irizarry et al., 2020; Kuniishi et al., 2022).

In contrast to what we saw in males, the current study found that post-weaning social isolation decreased social preference in adult female mice. One factor that may be mediating these effects is the impact of social hierarchy. While social dominance hierarchies alter the impact of social reward in males such that subordinate males find social interaction less rewarding, this is not the case in females (Cross et al., 2024). Therefore, the differences in social interaction seen in the group housed animals may be mediated, in part, by social hierarchy and the lack of hierarchical experience may increase social reward in males.

Our work adds to the growing literature examining the role of isolation stress on the long-term behavioral impairments in social behavior in females (Hermes et al., 2011; Tanaka et al., 2019; Wang Z.-J. et al., 2022). The sex differences seen in the current study are consistent with the sex-specific outcomes of social stress in humans (Kessler et al., 2012; Altemus et al., 2014; Laman-Maharg and Trainor, 2017; Liu et al., 2020) and they point toward different neurological sequelae in response to social isolation. In fact, adolescent social isolation leads to sex-specific alterations in gene expression within the prefrontal cortex, nucleus accumbens, and ventral tegmental area (Walker et al., 2022). Overall, our data here suggests that social isolation during adolescence leads to opposing behavioral effects during young adulthood in males and females and provides further support for sex differences in the mechanisms driving social stress induced behavioral alterations.

### 4.2 Post-weaning social isolation stress does not impact social behavior during adolescence

Given the literature on stress sensitivity during adolescence, we would expect to see post-weaning social isolation stress led to social behavioral impairments during this time. Interestingly, we did not observe this effect within the present study.

We found that socially isolated males and females did not display a significant difference in their social preference when tested during adolescence compared to their group-housed controls. While this may seem contradictory to previous work, few studies exploring the effects of post-weaning social isolation stress have tested the animal's social interaction behavior during the adolescent time point. Further this is consistent with work demonstrating increased “resilience” to stress during adolescence can manifest as alterations later in adulthood (Pantoja-Urbán et al., 2024).

With these results seen in the present study, we expand upon the importance of examining social behavior at various timepoints to better the understanding of behavioral changes produced by isolation stress, as well as understanding the periods of development that are most sensitive to the stress exposure. Although adolescence has been established as a critical period of development in its relation to stress sensitivity, there may be greater effects on long-term brain development and behavior, with increasing prevalence during adulthood. With this, more research is needed investigating the impact of early social isolation stress on adolescent social behavior in both males and females (Lukkes et al., 2009; Eiland and Romeo, 2013; Walker D. M. et al., 2019).

### 4.3 Adolescent minocycline treatment reverses decrease in social preference in young adult females following post-weaning social isolation

It is well-established that early life stress leads to alterations in brain structure and function, which have the capacity to induce behavioral changes. Of the brain structures that are altered, microglia exhibit increased vulnerability to psychosocial stress exposure (Tynan et al., 2010; Wohleb et al., 2011; Hinwood et al., 2013; Bollinger et al., 2016; Gildawie et al., 2020; Brenhouse, 2023; Biltz et al., 2024). Social stress during adolescence leads to changes in microglial structure and function (Cao et al., 2021; Lee et al., 2022; Singaravelu et al., 2022; Wang Y. et al., 2022; Xu et al., 2022), with changes playing an important role in mediating social behavior (Catale et al., 2020; Piirainen et al., 2021). Further, the impact of stress during adolescence on microglia is often sex-specific, with females exhibiting more stress-induced increases in microglial complexity than males (Bekhbat et al., 2021). Although highly complex, ramified microglia have long been considered quiescent, following chronic stress, microglia exhibit a hyper-ramified morphological state that is correlated with stress-induced behavioral alterations (Walkera et al., 2013; Walker D. J. et al., 2019; Piirainen et al., 2021). One such behavioral alteration seen following stress-induced microglial activation, is decreased sociability (Wohleb et al., 2012, 2014; Ramirez et al., 2015; Stein et al., 2017). Administration of minocycline, an antibiotic with anti-inflammatory properties, attenuates the over-activation of microglia (Hinwood et al., 2013; Kobayashi et al., 2013; Catale et al., 2020). Following stress exposure, minocycline administration can reverse the microglial hyper-ramification and, in turn, reverse stress-induced behavioral impairments (Hinwood et al., 2013; Schmidtner et al., 2019; Catale et al., 2020).

Consistent with this previous work, we showed that adolescent minocycline treatment was able to reverse the effect of post-weaning social isolation stress on social behavior in the females during young adulthood. This effect of minocycline seen here is consistent with the literature reflecting minocycline's ability to attenuate behavioral deficits following exposure to a stressor (Wang et al., 2018; Schmidtner et al., 2019). Our work contributes to the growing research demonstrating minocycline's role in alleviating behavioral deficits through the inhibition of hyper-ramified microglia. Of note, although minocycline does inhibit microglial activation, it is a tetracycline antibiotic that has other effects that are not specific to microglia, including actions on the gut microbiome (Kobayashi et al., 2013; Aronson, 2016; Schmidtner et al., 2019). Therefore, although our work suggests that microglia are playing a role in social behavior following stress, future studies using more specific manipulations of microglia are needed.

### 4.4 Adolescent minocycline treatment diminishes social preference in males during adolescence while not impacting behavior in young adulthood

Here we found that both the adolescent group-housed and socially isolated males exposed to adolescent minocycline treatment displayed a decrease in social preference compared to their baseline controls. While this result may seem to conflict with previous findings demonstrating minocycline's protective effects following behavior impairments, administration of the drug has shown to lead to adverse reactions in some cases, potentially attributed to antibiotic induced shifts in the microbiota (Aronson, 2016; Catale et al., 2020; Carson et al., 2023). In addition to the potential aversive effects, minocycline treatment has also shown to lead to reductions in locomotor behavior in rodents (Diguet et al., 2004a,b; Chen et al., 2009). While we did not see any differences in the locomotor behavior during the social interaction test in the current study, there may be adverse effects that easily visible. Taken together, this may explain why the adolescent males of the present study display a decrease in social preference, as the treatment may have led to the animals experiencing adverse reactions to the drug, leading to a decrease in locomotor behavior, further impacting their sociability.

### 4.5 Adolescent minocycline treatment leads to sex-specific alterations in social behavior

Taken together, we find that the effects of adolescent minocycline administration are sex specific. While the majority of preclinical studies that have examined the impact of stress on neuroimmune signaling have examined only male animals (Tynan et al., 2010; Hinwood et al., 2012; Magalhães et al., 2024), human subjects research suggests that women may be particularly vulnerable to chronic stress exposure, including social isolation (Liu et al., 2020). Preclinically, chronic stress often leads to sex specific alterations in neuroimmune signaling (Deak et al., 2015; Bekhbat and Neigh, 2018a,b; Bekhbat et al., 2021). During development, microglial colonization differs across sex, with males exhibiting higher levels of microglial colonization early in life (PND4) and this flipping at PND30 to females exhibiting more microglia maintained in an activated state than males (Schwarz et al., 2012). Therefore, female mice may be more vulnerable to manipulations the alter neuroimmune function, like social isolation, during this time period whereas male mice would be more vulnerable earlier. This is supported by work demonstrating that during early postnatal development, microglial phagocytosis plays a critical role in the development of social play behavior in males but not females (Kopec et al., 2018; VanRyzin et al., 2022). The current study supports a role for microglia in adolescence in shaping adult female social behavior as inhibiting microglia with minocycline administration during adolescence blocked the ability of social isolation to disrupt social behavior, whereas the augmented social interaction seen in male mice following social interaction may not be driven by microglial function.

## 5 Conclusions

Overall, the current study has shown that post-weaning social isolation stress leads to a sex-specific effect on sociability during young adulthood, with the socially isolated males expressing an increase in social behavior while the females express a decrease. Furthermore, when exposed to adolescent minocycline treatment, we find that during adolescence, the male's experience a decrease in their social behavior, yet this does not carry over into young adulthood as they show no effect on social behavior at this timepoint. Although, the females express the opposite, showing no effect on their social behavior during adolescence yet an increase in social behavior during young adulthood. Our results seen here further support previous work highlighting minocycline's ability to attenuate behavioral deficits following stress exposure. This suggests that minocycline may be a potential treatment method for behavioral impairments following early life stress.

## Data Availability

The raw data supporting the conclusions of this article will be made available by the authors, without undue reservation.
